# Ginsenoside Rb1 Prevents Oxidative Stress-Induced Apoptosis and Mitochondrial Dysfunction in Muscle Stem Cells via NF-*κ*B Pathway

**DOI:** 10.1155/2022/9159101

**Published:** 2022-11-24

**Authors:** Wenxi Dong, Wenhao Chen, Hongbo Zou, Zile Shen, Dingye Yu, Weizhe Chen, Haojie Jiang, Xialin Yan, Zhen Yu

**Affiliations:** ^1^Department of Gastrointestinal Surgery, The First Affiliated Hospital of Wenzhou Medical University, Wenzhou, Zhejiang, China; ^2^Department of Gastrointestinal Surgery, Shanghai Tenth People's Hospital, School of Medicine, Tongji University, Shanghai, China; ^3^Department of Gastrointestinal Surgery, People's Hospital of Deyang City, Deyang, Sichuan, China; ^4^Department of General Surgery, Huadong Hospital, Fudan University, Shanghai, China; ^5^Department of Colorectal Anal Surgery, The First Affiliated Hospital of Wenzhou Medical University, Wenzhou, Zhejiang, China

## Abstract

Sarcopenia, featured by the progressive loss of skeletal muscle function and mass, is associated with the impaired function of muscle stem cells (MuSCs) caused by increasing oxidative stress in senescent skeletal muscle tissue during aging. Intact function of MuSCs maintains the regenerative potential as well as the homeostasis of skeletal muscle tissues during aging. Ginsenoside Rb1, a natural compound from ginseng, exhibited the effects of antioxidation and against apoptosis. However, its effects of restoring MuSC function during aging and improving age-related sarcopenia remained unknown. In this study, we investigated the role of Rb1 in improving MuSC function and inhibiting apoptosis by reducing oxidative stress levels. We found that Rb1 inhibited the accumulation of reactive oxygen species (ROS) and protected the cells from oxidative stress to attenuate the H_2_O_2_-induced cytotoxicity. Rb1 also blocked oxidative stress-induced apoptosis by inhibiting the activation of caspase-3/9, which antagonized the decrease in mitochondrial content and the increase in mitochondrial abnormalities caused by oxidative stress via promoting the protein expression of genes involved in mitochondrial biogenesis. Mechanistically, it was proven that Rb1 exerted its antioxidant effects and avoided the apoptosis of myoblasts by targeting the core regulator of the nuclear factor-kappa B (NF-*κ*B) signal pathway. Therefore, these findings suggest that Rb1 may have a beneficial role in the prevention and treatment of MuSC exhaustion-related diseases like sarcopenia.

## 1. Introduction

Muscle stem cells (MuSCs), also known as satellite cells, proximal to myofiber basal lamina, have the ability of differentiation and proliferation. After skeletal muscle injury, normally quiescent MuSCs are activated and further differentiate to contribute to the regeneration of muscle tissue and maintain the homeostasis of skeletal muscle. As oxidative stress induces damage and inflammation, the regenerative capacity of skeletal muscle is largely dependent on its self-renewal ability through activating and differentiating the MuSCs [1].

Accumulating evidences have indicated that dysfunction of MuSCs was detected in various kinds of myopathies, such as Duchenne muscular dystrophy and disused muscular atrophy [2–4]. Notably, age-related muscle disorders, including sarcopenia, were featured by the exhausted MuSC pool and senescent MuSCs with reduced function [5–7]. The impairment of the MuSC population or function made it vulnerable to free-radical stress and delayed the recovery from damage. Mechanically, senescent MuSCs suffered a higher level of oxidative stress and are easily removed through the apoptosis process or entered an irreversible state of quiescence, which may due to the increased systematic inflammation and extrinsic factors in circulation of old individuals [8, 9]. Besides, productions of oxidative stress and inflammatory mediators would promote ER stress and mitochondrial dysfunction [10, 11], which would accelerate the process of apoptosis of MuSCs during aging. Recent studies focused on the antioxidative effects of several compounds to restore the regenerative capacity of MuSCs in senescent skeletal muscle [12, 13]. Unfortunately, the effective pharmacological treatment for rejuvenating MuSCs and improving geriatric skeletal muscle function remained elusive.

Ginsenoside Rb1 is a bioactive component extracted from ginseng that has antioxidant, anti-inflammation, and antisenescence effects [14, 15]. A recent study reported that ginsenoside could promote myogenic differentiation via Akt/mTOR signaling [16]. However, whether ginsenoside Rb1 could alleviate oxidative damage of MuSCs or rejuvenate MuSCs in senescent skeletal muscle is unknown. In this study, we investigated the role of Rb1 in improving MuSC function and inhibiting apoptosis by reducing oxidative stress levels. The underlying mechanism indicating the recovery of mitochondrial dysfunction and activation of the NF-*κ*B pathway was further elucidated. Moreover, this study also evaluated whether Rb1 contributed to the maintenance of homeostasis and the MuSC population in senescent skeletal muscle tissue in vivo. Such effects would be indicative of Rb1 having a potential role in treating age-related sarcopenia.

## 2. Materials and Methods

### 2.1. Cell Culture and Treatment

C2C12 mouse myoblasts, purchased from the American Type Culture Collection (Manassas, VA, USA), were cultured in a growth medium (Dulbecco's modified Eagle's medium (DMEM) with 10% FBS) under a humidified atmosphere with 5% CO_2_ at 37°C in an incubator. Ginsenoside Rb1 (purity ≥ 99%, Tongtian Biotechnology, Shanghai, China) was dissolved in DMSO as a storage liquid with a concentration of 100 mM, which was diluted with complete DMEM medium to adjust the final treatment concentration before usage in the experiments. When cell density reached 60-70%, C2C12 mouse myoblasts were, respectively, pretreated with different concentrations of ginsenoside Rb1 (20 *μ*M and 40 *μ*M) for 24 h and then treated with 1000 *μ*M H_2_O_2_ for 6 h.

### 2.2. Cell Viability Analysis

C2C12 mouse myoblasts, which were seeded in 96-well plates (5 × 10^3^ cells/well) and cultured for 24 h, were treated with H_2_O_2_ and Rb1 as mentioned above. The cell viability was determined with the Cell Counting Kit-8 (CCK-8, Yeason, China). After being washed with PBS, cells were incubated with the CCK-8 working solution at 37°C in the dark for 1-2 h, and then, optical density (OD) values at the wavelength of 450 nm were measured with a microplate reader (Bio-Rad Laboratories Inc., Hercules, CA, USA).

### 2.3. ATP Content Detection

C2C12 mouse myoblasts in 6-well plates were fully lysed with a lysis buffer, and the supernatant was collected by centrifugation (12000 g, 4°C, 5 min). ATP content in each sample was examined using an Enhanced ATP Assay Kit (Beyotime, Beijing, China) by a microplate reader (Bio-Rad Laboratories Inc., Hercules, CA, USA) and normalized by protein concentration which was quantified by a BCA protein concentration kit (Thermo Fisher Scientific, Waltham, MA, USA).

### 2.4. Colony-Forming Assay

After the treatments above, C2C12 mouse myoblasts in each group were digested with EDTA and seeded in 6-well plates (1200 cells/well). The cells were cultured at 37°C in an incubator for 12-14 days, until visible colonies were observed. After being washed with PBS for three times, the colonies were fixed with 4% PFA Fix Solution and then stained with Crystal Violet Staining Solution. The size and number of colonies in each well were counted.

### 2.5. Western Blot Assay

C2C12 mouse myoblasts in different groups were lysed using RIPA containing protease and phosphatase inhibitor cocktail (Roche, Indianapolis, IN, USA). After centrifugation, the total proteins were extracted, and the concentrations were measured by BCA assay. An equal amount of protein was separated by SDS/PAGE. Membranes were blocked with 5% nonfat milk at 4°C overnight and probed with primary antibodies. The primary antibodies used in this study are presented in Table S1. The membranes were then incubated with the secondary antibodies (room temperature, 60 min), and membranes were detected by either the ECL Western blot detection system or the Odyssey system.

### 2.6. Confocal Laser Scanning Microscopy

The C2C12 mouse myoblasts were cultured in confocal dishes and stained with JC-1 (JC-1 Mitochondrial Membrane Potential Assay Kit, Yeason, China), MitoTracker (MitoTracker Red CMXRos, Beyotime, Beijing, China), MitoSOX (MitoSOX Red Mitochondrial Superoxide Indicator, Yeason, Shanghai, China), or DCFH-DA (ROS Assay Kit, Beyotime, Beijing, China) according to the manufacturer's protocol. The fluorescent image was acquired, and the red/green/blue fluorescence signal was separated using confocal laser scanning microscopy (CLSM) in an epifluorescence mode with laser and filter of the 488/535/595 nm line. Fluorescence intensity of the images was analyzed using ImageJ software, and data was reported as a mean gray value, which was calculated by integrated density divided by area.

### 2.7. Flow Cytometry

C2C12 mouse myoblasts in each group were digested by trypsin solution without EDTA and stained with Annexin V-FITC/PI (apoptosis detection kit, BD Biosciences, CA, USA), DCFH-DA, or JC-1 according to the manufacturer's protocol. Subsequently, the results determined by a flow cytometer (Becton, Dickinson, Franklin Lakes, NJ, USA) were analyzed by FlowJo software (version 7.6.1; FlowJo LLC).

### 2.8. Live/Dead Assay by Calcein-AM/PI Double Staining

C2C12 mouse myoblasts cultured on a 12-well plate were digested and then collected by centrifugation. The cells were stained with a Calcein-AM/PI Double Stain Kit (Yeason, Shanghai, China) according to the manufacturer's protocol. The green fluorescence produced by living cells and the red fluorescence produced by dead cells were detected simultaneously under a fluorescence microscope using excitation light of different wavelengths with an inverted fluorescence microscope (Leica Microsystems, Wetzlar, Germany).

### 2.9. Transmission Electron Microscopy (TEM)

The C2C12 mouse myoblasts were digested and collected by centrifugation. The cells were subsequently fixed with 2.5% glutaraldehyde for 30 min at room temperature and preserved at 4°C for 24 h. After being dehydrated in an acetone dilution series, the samples were embedded in an epoxy resin. Ultrathin sections (80 nm) were obtained with an Ultracut UCT microtome (Leica UC7, Leica, Germany) and observed with a transmission electron microscope (HT7700, Hitachi, Japan) at 80 kV. For each sample, the proportion of abnormal mitochondria (abnormal/total mitochondria) was measured in 3 randomly selected fields. Mitochondria were identified as abnormal if they have disrupted membranes, crista depletion, matrix dissolution, and vacuolization.

### 2.10. Quantitative RT-PCR Analysis

Total RNA of C2C12 mouse myoblasts were extracted with a TRIzol® reagent (Life Technologies, Carlsbad, USA), and total RNA was treated with a PrimeScript RT reagent kit to erase gDNA (Takara, Shiga, Japan). To obtain cDNA, 500 *μ*g of total RNA was used for reverse-transcription. Real-time quantitative PCR was performed by QuantStudio™ Dx real-time PCR instrument (Thermo Fisher Scientific, Singapore) with TB Green® premix Ex Taq™ II (Takara, Shiga, Japan). The primers of the real-time PCR analysis are listed in Table S2.

### 2.11. Animals and Treatments

Young (about 6-month-old) and old (about 20-month-old) male C57BL/6 mice were housed in SPF facilities in a 12 h light-dark cycle room and had access to the standard rodent diet and water ad libitum. The old mice were intraperitoneally injected with either Rb1 (20 mg/kg body weight, Rb1 group) or normal saline (old control group) once a day for 4 weeks, and the young mice were intraperitoneally injected with normal saline (young control group). Rb1 was solubilized in normal saline with a final concentration of 4 mg/ml for injection. All research protocols were approved by the Institutional Animal Committee of Tongji University and conformed to the Guide for the Care and Use of Laboratory Animals.

### 2.12. Sample Collection

After 24 h of the last treatment, these mice were sacrificed by cervical dislocation, and the gastrocnemius (GA) and tibialis anterior (TA) in different groups were dissected and weighed quickly. The skeletal muscle samples were then snap-frozen in liquid nitrogen and stored at -80°C or fixed 4% paraformaldehyde which were then embedded in paraffin and sectioned for dihydroethidium (DHE) staining and immunofluorescence staining.

### 2.13. DHE Staining

After the mice were sacrificed, TA were frozen in -80°C for DHE staining. 8 *μ*m frozen sections were incubated with a DHE working solution (Merck, Germany) in the dark for 45 min and viewed with a fluorescence microscope (Leica, Germany). Fluorescence intensity of the images was analyzed using ImageJ software, and data was reported as mean gray value, which was calculated by integrated density divided by area.

### 2.14. TUNEL Assay

TUNEL (terminal deoxynucleotidyl transferase dUTP nick end labelling) fluorescent detection kit was used to detect apoptotic cells on the paraffin-embedded GA sections, according to the manufacturer's instructions. Immunofluorescence was viewed with a fluorescence microscope (Leica, Germany). TUNEL-positive cells were counted, and data was reported as a percentage of TUNEL-positive muscle cells, which was calculated by counting the total number of TUNEL-positive nuclei divided by the total nuclei.

### 2.15. Immunofluorescent Staining

Immunofluorescent staining was performed on GA transverse sections using the paired box 7 (PAX7, a marker protein of MuSCs) and dystrophy antibodies. The fixed GA transverse sections were incubated with primary antibodies (PAX7 and dystrophy) at 4°C overnight on a shaker. Following incubation with the secondary antibodies at room temperature for 1 h, all the samples were later counterstained with 4′-6-diamidino-2-phenylindol (DAPI) for 1 h. Data was reported as a percentage of PAX7-positive cells, which was calculated by counting the total number of PAX7-positive nuclei divided by the total nuclei.

### 2.16. Molecular Docking and Bibliometric Analysis

The molecular docking of NF-*κ*B and ginsenoside Rb1 was achieved using AutoDock Tools 1.5.6, Pymol 2.3, AutoDock Vina 1.1.2, and DS Visualizer. The publications containing “ginsenoside Rb1” as the keywords were extracted from Web of Science and imported to VOSviewer to perform visual analysis.

### 2.17. Statistical Analysis

Continuous variables were compared by one-way ANOVA. *P* value < 0.05 was considered statistically significant. Data were analyzed and presented as mean ± SEM (^∗^*P* < 0.05: control vs. other groups; ^&^*P* < 0.05: H_2_O_2_ vs. Rb1 groups) by SPSS Statistics software (version 23.0 IBM, NY, USA) and GraphPad Prism software (version 7.6). All in vitro experiments were performed with a minimum of three independent trials. All in vivo experiments were performed with a minimum of three animals per group.

## 3. Results

### 3.1. Ginsenoside Rb1 Suppressed H_2_O_2_-Induced C2C12 Cell Cytotoxicity

**Figure 1:**
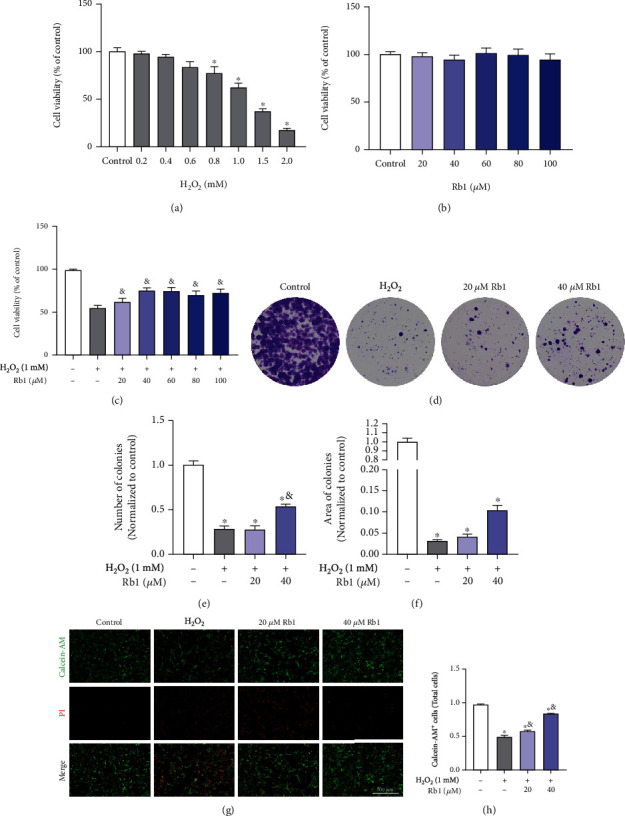
Effects of ginsenoside Rb1 on the H_2_O_2_-induced damage in C2C12 myoblasts. (a–c) Cell activity was assessed by the CCK-8 assay. (a) C2C12 myoblasts were treated with H_2_O_2_ (0, 200, 400, 800, 1000, 1500, and 2000 *μ*M) for 6 h. (b) C2C12 myoblasts were treated with Rb1 (0, 20, 40, 60, 80, and 100 *μ*M) for 24 h. (c) C2C12 myoblasts were pretreated with Rb1 (0, 20, 40, 60, 80, and 100 *μ*M) for 6 h and then treated with 1000 *μ*M H_2_O_2_ for 24 h. (d) Representative images of colony-forming assay of C2C12 myoblasts in different groups. (e, f) Quantitative analysis of number and area of colonies (*n* = 3 in each group). (g) Representative images of Calcein-AM/PI double staining of C2C12 myoblasts. (h) The percentage of PI+ cells in different groups (*n* = 3 in each group). Data show mean ± SEM (^∗^*P* < 0.05: control vs. other groups; ^&^*P* < 0.05: H_2_O_2_ vs. Rb1 groups).

To identify the effects of H_2_O_2_ and Rb1 on myoblasts, the results of the CCK-8 assay in Figure 1(a) presented no significant difference in cell vitality of C2C12 cells with Rb1 below concentrations of 1500 *μ*M. The vitality of C2C12 cells was reduced about 40% at 1000 *μ*M of H_2_O_2_ (Figure 1(b)). According to previous research, this H_2_O_2_ concentration was favorable to induce oxidative stress and was used for further study [17]. As is shown in Figure 1(c), Rb1 alleviated the H_2_O_2_-induced loss of cell vitality in C2C12 mouse myoblasts, and 20 *μ*M (lower dose) and 40 *μ*M of Rb1 (higher dose) were used in further experiments. Colony-forming assay demonstrated that Rb1 ameliorated the decrease in the size and number of colonies caused by H_2_O_2_ treatment (Figures 1(d) and 1(e)). In Figures 1(g) and 1(h), we observed the significantly reduced percentage of Calcein-AM-positive cells after H_2_O_2_ treatment, indicating that H_2_O_2_-induced oxidative damage led to severe cell death, while this effect was significantly alleviated by treatment with 40 *μ*M Rb1.

### 3.2. Ginsenoside Rb1 Reduced Intracellular ROS Level and Protects C2C12 Myoblasts against Apoptosis

**Figure 2:**
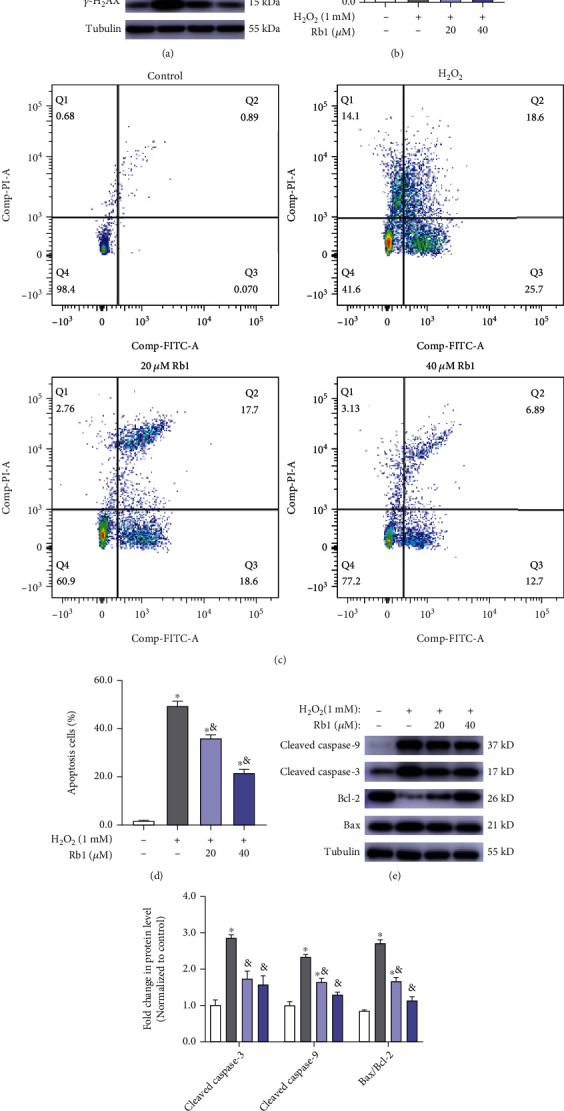
Ginsenoside Rb1 suppressed C2C12 myoblasts from apoptosis caused by H_2_O_2_. (a, b) Immunoblot analyses of *γ*-H2AX expression in C2C12 myoblasts of different groups. (c, d) Representative flow cytometry plots and quantitative analysis of cell apoptosis in different groups (*n* = 3 in each group). (e, f) Immunoblot analyses of apoptosis marker genes (cleaved caspase-3/9, Bax, Bcl-2) in C2C12 myoblasts of different groups. Data show mean ± SEM (^∗^*P* < 0.05: control vs. other groups; ^&^*P* < 0.05: H_2_O_2_ vs. Rb1 groups).

We further detected the protein expression of *γ*-H2AX, a marker of DNA damage during oxidative damage. The increase in protein level of *γ*-H2AX was determined in H_2_O_2_-treated cells and was blocked by Rb1 pretreatment (Figures 2(a) and 2(b)). Considering the pivotal role of *γ*-H2AX during apoptosis and oxidative damage [18, 19], Annexin V-FITC/PI double staining assay following flow cytometry was performed to validate their change in apoptosis. As shown in Figures 2(c) and 2(d), Rb1 incubation with H_2_O_2_ induced more than 40% cell apoptosis, and Rb1 improved the cell survival via reducing about 1/3-1/2 cell apoptosis caused by H_2_O_2_. The expressions of apoptosis-related proteins were also detected. Increased expression of cleaved caspase-3/9 and Bax caused by H_2_O_2_ was reversed by treatment with Rb1 treatment. Conversely, Rb1 also restored the expression of antiapoptotic protein Bcl-2, as well as the Bax-Bcl-2 ratio (Figures 2(e) and 2(f)). These results indicated that Rb1 could alleviate apoptosis of C2C12 myoblasts caused by H_2_O_2_.

**Figure 3:**
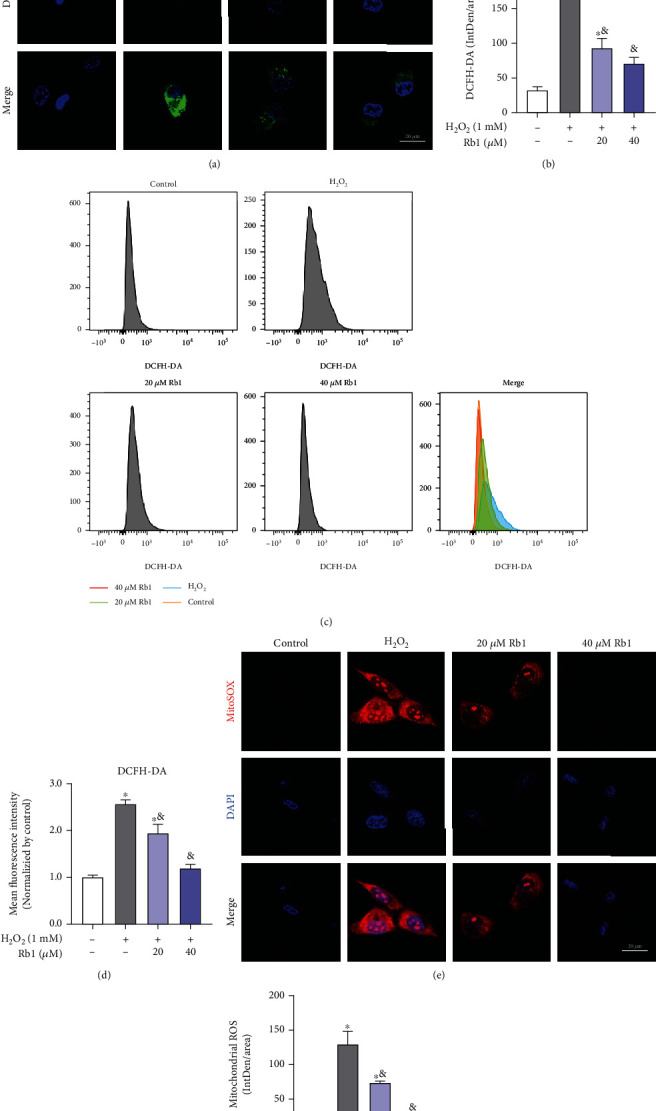
Ginsenoside Rb1 reduced the levels of ROS elevated by H_2_O_2_. (a, b) Representative laser confocal images of DCFH-DA staining of C2C12 myoblasts in different groups (*n* = 3 in each group). (c, d) Representative flow cytometry plots and quantitative analysis of DCFH-DA staining (*n* = 3 in each group). (e, f) Representative laser confocal images of MitoSOX staining of C2C12 myoblasts in different groups. Data shows mean ± SEM (^∗^*P* < 0.05: control vs. other groups; ^&^*P* < 0.05: H_2_O_2_ vs. Rb1 groups).

To confirm the effects of Rb1 on regulating the ROS level in myoblasts, the DCFH-DA staining was performed. As shown in Figures 3(a) and 3(b), the C2C12 cells exposed to H_2_O_2_ display significantly higher fluorescence intensity than those of the other groups, while the lower fluorescence intensity following Rb1 pretreatments indicates its effect on reducing the ROS level and oxidative stress. These results were also confirmed by flow cytometry in Figures 3(c) and 3(d). Mitochondria are the major intracellular organelles of ROS production and the main target of ROS-induced damage [20, 21]. The MitoSOX staining indicated that there was a significant increase in mitochondrial ROS level in cells treated with H_2_O_2_, which pretreatment with Rb1 decreased (Figures 3(e) and 3(f)). Taken together, these results suggested that Rb1 could mitigate oxidative injury of C2C12 cells by decreasing the intracellular ROS level to protect myoblasts against apoptosis.

### 3.3. Ginsenoside Rb1 Alleviated Oxidative Injury by Improving Mitochondrial Function In Vitro

**Figure 4:**
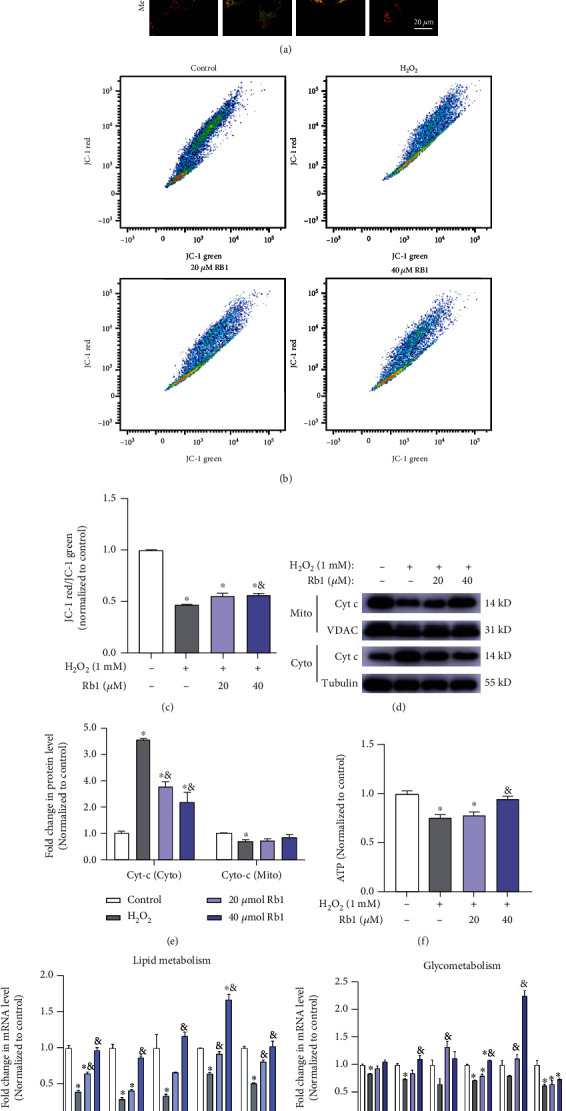
Ginsenoside Rb1 alleviated the H_2_O_2_-induced mitochondrial dysfunction. (a) Representative images of JC-1 staining of C2C12 myoblasts in different groups. (b, c) Representative flow cytometry plots and quantitative analysis of JC-1 staining (the rate of mean fluorescence intensity of JC-1 red/JC-1 green, normalized by control, *n* = 3 in each group). (d, e) Immunoblot analyses of cytochrome c in cytosol and mitochondria from C2C12 myoblasts in different groups. (f) ATP content was normalized by total protein content in different groups and then normalized to control. (g, h) Relative mRNA expression levels of the genes related to lipid metabolism (Cd36, Cpt1a, Cpt1b, Hadha, Hadhb) and Glysis (Eno3, Glut4, Gpd1, Hk1, Hk2, Pgk1). Data show mean ± SEM (^∗^*P* < 0.05: control vs. other groups; ^&^*P* < 0.05: H_2_O_2_ vs. Rb1 groups).

Decrease in mitochondrial membrane potential (MMP) severely impaired the mitochondrial function and also triggered apoptosis [22]. The JC-1 aggregate/monomer ratio formation was calculated to demonstrate the effects of oxidative injury on MMP alterations. The MMP of C2C12 myoblasts was reduced by H_2_O_2_, while Rb1 treatment improved H_2_O_2_-induced loss of MMP (Figures 4(a)–4(c)). Similarly, the release was observed for CytC from the mitochondria to the cytoplasm after oxidative injury caused by H_2_O_2_, which was improved by Rb1 pretreatment (Figures 4(d) and 4(e)).  Figure 4(f) shows that Rb1 significantly restored H_2_O_2_-induced loss of total ATP content in C2C12 cells, accompanied with the increasing mRNA expression of genes concerning lipid metabolism (CD36, Cpt1a, Cpt1b, Hadha, and Hadhb) and glycometabolism (Eno3, Glut4, Hk1, Hk2, and Gpd1) processes (Figures 4(g) and 4(h)). Thus, these data indicate that Rb1 maintained normal MMP levels and thereby reduced oxidative injury-induced mitochondrial dysfunction.

### 3.4. Ginsenoside Rb1 Promoted Mitochondrial Biogenesis and Quality Control following H_2_O_2_-Induced Oxidative Injury

**Figure 5:**
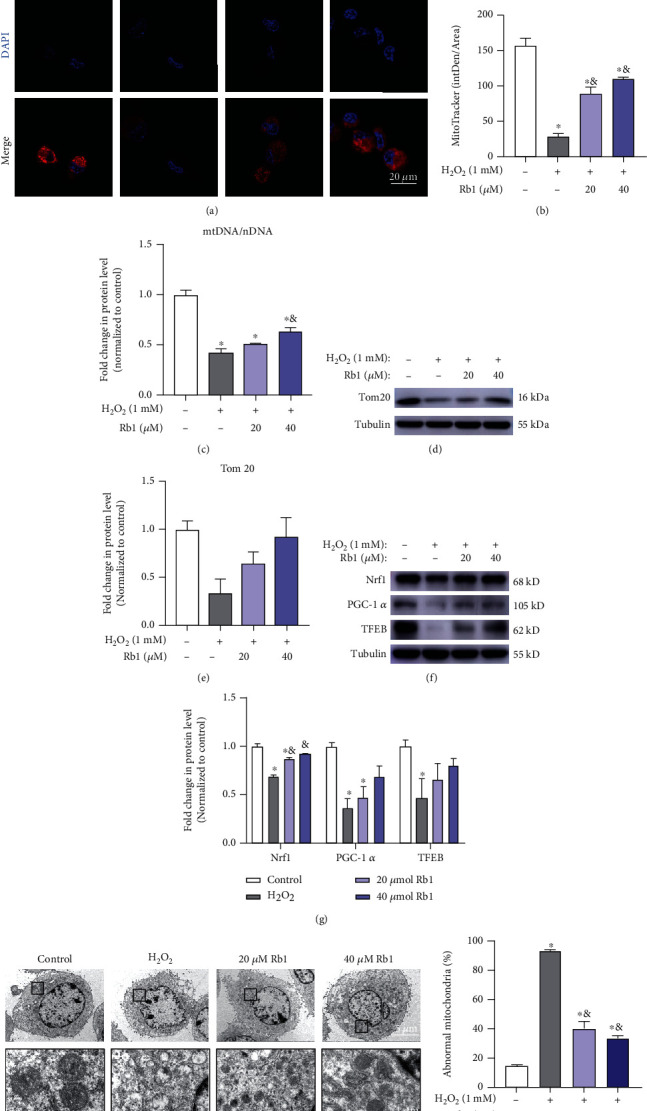
Ginsenoside Rb1 attenuated the decrease of mitochondrial content by promoting mitochondrial biogenesis. (a, b) Representative images of MitoTracker staining of C2C12 myoblasts in different groups. (c) Relative mRNA expression levels of mitochondrial DNA (normalized by nuclear DNA, *n* = 3 in each group). (d, e) Immunoblot detection of Tom20. (f, g) Immunoblot analyses of the genes related to mitochondrial biogenesis (Nrf1, PGC-1*α*, TFEB). (h) Representative TEM images of C2C12 myoblasts from different groups. (i) The percentage of abnormal mitochondria in different groups. Data show mean ± SEM (^∗^*P* < 0.05: control vs. other groups; ^&^*P* < 0.05: H_2_O_2_ vs. Rb1 groups).

Mitochondria are highly dynamic organelles that respond to the redox status and energy demands of cells. To further investigate the state of mitochondria in each group, MitoTracker staining was performed and visualized by confocal microscopy. As shown in Figures 5(a) and 5(b), C2C12 myoblasts exposed to H_2_O_2_ display the lowest fluorescence intensity compared to other groups, while pretreatment with Rb1 reversed the effects of H_2_O_2_, indicating its effects of increasing mitochondrial content. These results were also verified by the detection of mitochondrial DNA copy number by qPCR (Figure 5(c)) and the expression of mitochondrial membrane protein translocase of the outer membrane 20 (Tom20) by Western blot (Figures 5(d) and 5(e)). The Rb1-induced increases in the expression of the nuclear respiratory factor 1 (Nrf1), peroxisome proliferator-activated receptor-gamma coactivator-1alpha (PGC-1*α*), and transcription factor EB (TFEB) proteins that are markers of mitochondrial biogenesis document the effectiveness of such a treatment (Figures 5(f) and 5(g)). Ultrastructural analysis by TEM demonstrated damaged or swollen mitochondria with disordered cristae in the H_2_O_2_ group, with a significantly higher percentage of abnormal mitochondria compared to the control group. Moreover, Rb1 treatment seems to ameliorate the H_2_O_2_-induced mitochondrial injury to maintain the fitness of mitochondria in C2C12 myoblasts (Figures 5(h) and 5(i)). Taken together, these effects demonstrate the effectiveness of Rb1 in improving both mitochondrial biogenesis and quality control during oxidative injury.

### 3.5. Ginsenoside Rb1 Inhibited NF-*κ*B Signaling Pathway to Alleviate Oxidative Injury in Myoblasts

**Figure 6:**
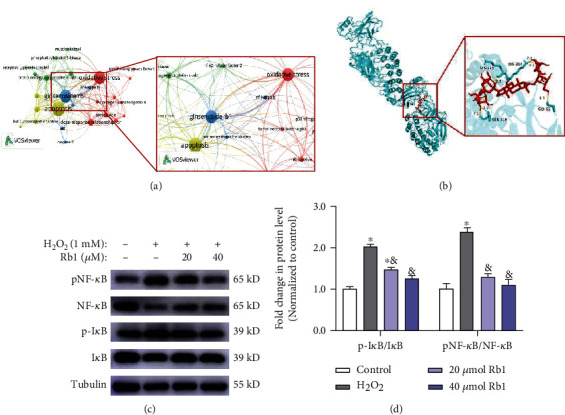
Ginsenoside Rb1 inhibited the activation of NF-*κ*B pathway. (a) Bibliometric analysis of the keywords in publications of ginsenoside Rb1 (cooccurrence of keywords). (b) Molecular docking diagram: molecular models of the binding of ginsenoside Rb1 with NF-*κ*B (-7.77 kcal/mol). (d, e) Immunoblot analyses of the genes related to NF-*κ*B pathway (NF-*κ*B, pNF-*κ*B, I*κ*B, pI*κ*B). Data show mean ± SEM (^∗^*P* < 0.05: control vs. other groups; ^&^*P* < 0.05: H_2_O_2_ vs. Rb1 groups).

To clarify the mechanisms of the beneficial effects of ginsenoside Rb1 in myoblasts, we searched studies related to ginsenoside Rb1 and processed them with the VOSviewer software. Among all the pathways that may interact with Rb1, we identified that NF-*κ*B signaling exerts the closest association with the keywords including apoptosis, ginsenoside Rb1, and oxidative stress (Figure 6(a)). It is reported that the activation of the NF-*κ*B pathway would promote inflammation and blunt the function of MuSCs [23]. Moreover, we performed the molecular docking simulation of the NF-*κ*B protein via AutoDock software, which demonstrated that Rb1 could bind to NF-*κ*B to form a stable structure (docking score: -7.77 kcal/mol, Figure 6(b)). The NF-*κ*B signaling pathway-related marker proteins (NF-*κ*B, I*κ*B, p-I*κ*B, and p-NF-*κ*B) were then detected by Western blot. And the expression levels of p-IKB and p-NF-*κ*B were increased in myoblasts of the H_2_O_2_ group and decreased following Rb1 pretreatment, indicating the inhibition of NF-*κ*B signaling by Rb1 (Figures 6(c) and 6(d)). These results suggest that the effects of Rb1 in alleviating oxidative stress may result from the interaction of Rb1 and NF-*κ*B to suppress the NF-*κ*B pathway in myoblasts.

### 3.6. Ginsenoside Rb1 Reduced the ROS Levels and Preserved the MuSC Pool in Senescent Skeletal Muscle In Vivo

**Figure 7:**
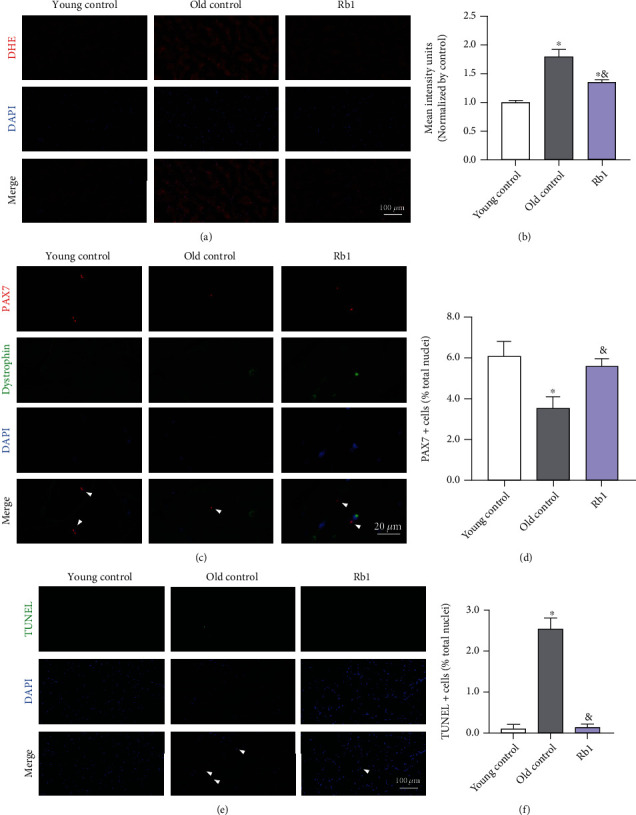
Ginsenoside Rb1 inhibited apoptosis and restored MuSC pool in senescent skeletal muscle tissue. (a) Representative images of the cross-sections of tibialis anterior (TA) by DHE staining. (b) Total ROS. (c) MuSCs in the cross-sections of gastrocnemius (GA) colabelled for DAPI, dystrophin, and PAX7. (d) Percentage of Pax7+ cells (relative to total DAPI+ nuclei). (e) Apoptosis cells in cross-sections of TA colabelled for DAPI and TUNEL. (f) Percentage of TUNEL+ cells (relative to total DAPI+ nuclei). Data show mean ± SEM (^∗^*P* < 0.05: control vs. other groups; ^&^*P* < 0.05: H_2_O_2_ vs. Rb1 groups).

Increasing oxidative stress, a major characteristic of aging, has been implicated in a variety of age-related pathologies [24]. To clarify antioxidant effects of ginsenoside Rb1 in MuSC in vivo, we then treated old mice (about 22 months old) with Rb1 for 1 month. DHE staining was performed to detect the ROS levels in skeletal muscle. As shown in Figures 7(a) and 7(b), the ROS levels increased in senescent skeletal muscle compared to young skeletal muscle tissue, which was significantly reduced following Rb1 treatment. Immunofluorescent staining of DAPI and paired box 7 (PAX7), the marker protein of MuSCs, demonstrated the higher percentage of MuSCs (PAX7+/DAPI+) in the Rb1-treated group compared to the old control group, which suggests that the effects of Rb1 reduce losses in the number of MuSCs in senescent skeletal muscle (Figures 7(c) and 7(d)). The results shown in Figures 7(e) and 7(f) confirm that Rb1 treatment decreased the TUNEL+ cells compared with those in the old control group suggesting that apoptosis in the senescent skeletal muscle was suppressed by Rb1. All these data indicate that Rb1 blunted oxidative stress-induced losses in the MuSC pool by reducing ROS levels and inhibiting apoptosis to maintain the regenerative potential of senescent skeletal muscle tissue.

## 4. Discussion

**Figure 8:**
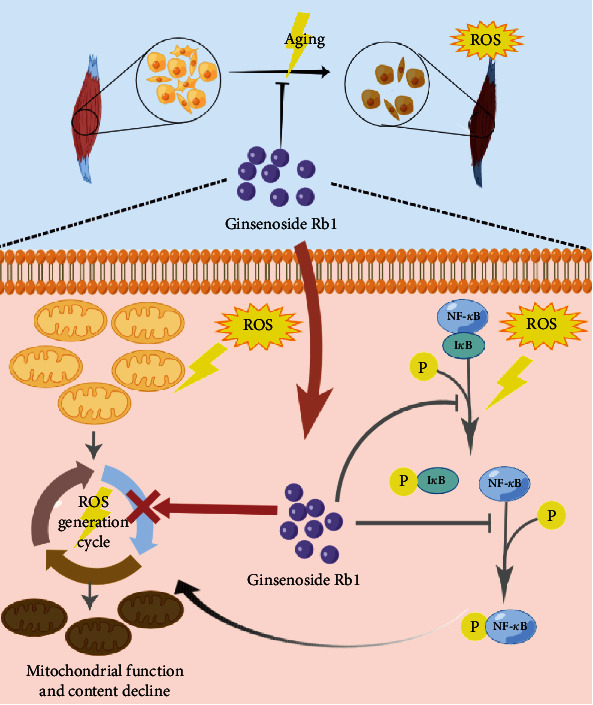
Schematic illustration of ginsenoside Rb1 protecting MuSCs from ROS damage and apoptosis. Ginsenoside Rb1 binds to the NF-*κ*B protein and inhibits the NF-*κ*B pathway from alleviating the oxidative damage and restoring the mitochondrial function in senescent MuSCs, which in turn improves MuSC function and maintains the skeletal muscle fitness during aging. This indicates ginsenoside Rb1 might be a potential pharmacological treatment for age-related sarcopenia. This figure was created using Figdraw.

Dysfunction or exhaustion of MuSCs commonly occurs in senescent skeletal muscle. This finding made it evident that maintaining the vitality as well as homeostasis of MuSCs is the key to cure age-related sarcopenia [25, 26]. In the current study, we demonstrated the beneficial role of ginsenoside Rb1 in relieving oxidative stress of MuSCs. Our results indicate that ginsenoside Rb1 can restore the content and function of mitochondria by improving mitochondrial biogenesis and inhibit the apoptosis both in vitro and in vivo, contributing to the preservation of MuSCs in senescent skeletal muscle. It was further confirmed that ginsenoside Rb1 may interact with the NF-*κ*B protein to inhibit NF-*κ*B signaling and reduce the ROS levels in MuSCs (Figure 8). To the best of our knowledge, this study is the first to investigate the effects of ginsenoside Rb1 on inhibiting apoptosis signaling and restoring mitochondrial dysfunction of MuSCs to maintain the fitness of senescent skeletal muscle. We believe that Rb1 can serve as a potential pharmacological treatment for age-related sarcopenia.

Recent studies elucidated the pathological features of senescent skeletal muscle tissues, including fat infiltration, interstitial fibrosis, chronic inflammation, etc. [27–29]. Some of these pathological changes were also observed in several myopathies with impaired regenerative capacity of MuSCs [30, 31]. Moreover, the changes of interstitial tissues and the microenvironment indicate the increased oxidative stress during aging [32]. And the excessive activation of MuSCs by oxidative damage or ROS would eventually lead to the dysfunction and exhaustion of MuSCs in senescent skeletal muscle [33, 34]. With regard to treatments such as BST204 (a purified extract from herbs) and *β*-hydroxy-*β*-methylbutyrate (HMB) supplements, an intermediate product of leucine metabolism focused on myogenesis and improving function of myotubes to offer new treatments for ageing-associated muscle decline, their underlying mechanisms involve the AMPK*α*/Sirt1/PGC-1*α* and PI3K/Akt pathways [35–37], whereas currently, there is no approved pharmacological treatment for sarcopenia [38]. In our study, compared with the old control group, the ROS level as well as apoptosis was decreased while more MuSCs were observed in the Rb1-treated group. These results indicate that Rb1 may suppress apoptosis and preserve the satellite cell pool, which in turn suggests that it can potentially provide a treatment for sarcopenia.

Despite that the activation of MuSCs is partially dependent on ROS, the damage by ROS accumulation is well known, especially when the ROS level overwhelms antioxidative defenses in senescent tissues. On the other hand, excessive activation of MuSCs induced by ROS would ultimately lead to the exhaustion of MuSCs [39]. Mitochondria and their components were the primary targets of oxidative damage. In particular, the mitochondria underwent changed membrane potential, disrupted replication or transcription of mtDNA, and electron transport chain (ETC) abnormalities [40]. Therefore, mitochondrial dysfunction is regarded as the hallmarks of senescence. As a natural compound, ginsenoside Rb1 is able to act as an antioxidant, anti-inflammatory, and antiapoptosis in adipocytes and endotheliocytes [41–43], while few researches have investigated the positive effects of Rb1 on improving mitochondrial function of MuSCs in senescent muscle tissue [16]. Our data showed that Rb1 protected MuSCs from MMP loss and leakage of cytochrome c to maintain the fitness of mitochondria which would explain the improved ATP generation after pretreatment with Rb1 in Figure 4. Moreover, it is reported that mitochondria contribute to several cellular stress responses including apoptosis, and its outer membrane serves as a unique signaling platform for Bcl-2 protein-dependent apoptosis [44–46]. Thus, it is not surprising to find that Rb1 can also inhibit apoptosis of MuSCs by mediating the expression of apoptosis-related proteins (cleaved caspase-3/9, Bcl-2, and Bax) and increase the number of MuSCs in senescent skeletal muscle in vivo (Figures 2 and 7).

Accumulating evidences show that the overactivation of the NF-*κ*B pathway by the local inflammatory signals in skeletal muscle tissue impairs MuSCs [47]. In terms of geriatric skeletal muscle, the common activators of the NF-*κ*B pathway include chronic inflammation and higher levels of ROS or inflammatory factors (IL-1, IL-6, TNF-*α*, etc.) than those elicited from their younger counterpart [48]. In addition, I*κ*B and NF-*κ*B could mediate various signaling pathways including proliferation, apoptosis, and inflammation [49, 50]. A recent study also demonstrated that the application of pyrrolidine dithiocarbamate (PDTC), a NF-*κ*B inhibitor, provides a potential therapeutic strategy for TNF-*α*-induced muscle atrophy [51]. In this study, Rb1 alleviated H_2_O_2_-induced oxidative damage in MuSCs, which paralleled the reduced expression of p-NF-*κ*B and p-I*κ*B, implying the inhibition of the NF-*κ*B signaling pathway. AutoDock simulations confirmed an interaction between Rb1 and NF-*κ*B. Taken together, we conclude that Rb1 directly interacts with NF-*κ*B to alter downstream signaling events that promote antioxidant effects which in turn improve senescent skeletal muscle MuSC function.

## 5. Conclusions

Ginsenoside Rb1 improved the mitochondrial function of myoblasts and protected them against oxidative stress by inhibiting NF-*κ*B signaling. Rb1 also inhibited apoptosis of MuSCs and reduced the ROS levels in senescent skeletal muscle in vivo. Therefore, Rb1 could restore MuSC function to maintain skeletal muscle tissue homeostasis and serve as a pharmacological treatment for age-related sarcopenia.

## Data Availability

The data generated for this study are all included in the manuscript.

## Supplementary Materials

Supplementary Materials Table S1: primer antibodies used for Western blot in the study. Table S2: primer sequences for q-PCR analysis.
