# Supplementary material for: Ginsenoside Rb1 Prevents Oxidative Stress-Induced Apoptosis and Mitochondrial Dysfunction in Muscle Stem Cells via NF-κB Pathway
Source: Oxid Med Cell Longev. 2022 Nov 24;2022:9159101. doi: 10.1155/2022/9159101 (PMC9715322; doi:10.1155/2022/9159101)
Supplement: Supplementary Materials — Table S1: primer antibodies used for Western blot in the study. Table S2: primer sequences for q-PCR analysis. [file 9159101.f1.zip › Table S1.doc]

**Table S1.** Primer antibodies used for Western blot analysis in the study.

| **Antibody** | **Dilution ratio** | **Supplier** | **Catalogue Number** |
| --- | --- | --- | --- |
| Tom20 | 1:1000 | Cell Signaling Technology | 42406 |
| Tubulin | 1:2000 | Cell Signaling Technology | 2125 |
| VDAC-1 | 1:2000 | Abcam | ab15895 |
| γ-H2AX | 1:1500 | Abcam | ab26350 |
| cleaved caspase-9 | 1:1000 | Cell Signaling Technology | 9509 |
| cleaved caspase-3 | 1:1000 | Cell Signaling Technology | 9664 |
| Bcl-2 | 1:1000 | Abmart | T40056 |
| Bax | 1:1500 | Abmart | T40051 |
| Cytochrome C | 1:1000 | Abcam | Ab133504 |
| Nrf1 | 1:1500 | Cell Signaling Technology | 46743 |
| PGC-1α | 1:500 | Abcam | ab54481 |
| TFEB | 1:1000 | Abcam | ab220695 |
| pNF-κB | 1:1000 | Cell Signaling Technology | 3033 |
| NF-κB | 1:1000 | Cell Signaling Technology | 8242 |
| p-IκB | 1:1500 | Affinity | AF2002 |
| IκB | 1:1000 | Abcam | 4814 |
| PAX7 | 1:1000 | Affinity | AF7584 |
| dystophin | 1:1000 | Santa | Sc-47760 |
