# Supplementary material for: Ginsenoside Rb1 Prevents Oxidative Stress-Induced Apoptosis and Mitochondrial Dysfunction in Muscle Stem Cells via NF-κB Pathway
Source: Oxid Med Cell Longev. 2022 Nov 24;2022:9159101. doi: 10.1155/2022/9159101 (PMC9715322; doi:10.1155/2022/9159101)
Supplement: Supplementary Materials — Table S1: primer antibodies used for Western blot in the study. Table S2: primer sequences for q-PCR analysis. [file 9159101.f1.zip › Table S2.doc]

**Table S2. Primer sequences for q-PCR analysis and knockdown**

| **Gene** | **Forward primer** | **Reverse primer** |
| --- | --- | --- |
| CD36 | GACTGGGACCATTGGTGATGA | AAGGCCATCTCTACCATGCC |
| Cpt1a | CATCGTGAGTGGCGTCCTCTTTG | AGTGCTGTCATGCGTTGGAAGTC |
| Cpt1b | ACAGACTTGCTACAGCACCTC | CGTCGAGGATTCTCTGGAC |
| Eno3 | CGACACATCGCAGATCTTGC | CCGTTGATCACATTAAAGGCAG |
| Glut4 | TCTGTGGGTGGCATGATCTCT | CCAACACGGCCAAGACATTG |
| Gpd1 | CCCATGAGCGTGCTGATG | GTGATGCGAAAGTTGGGTGTCT |
| GADPH | TTCAACAGCAACTCCCACTCTT | TGGTCCAGGGTTTCTTACTCC |
| HK1 | GAGGTCTACGACACCCCAGA | GAAGTCTCCGAGGCATTCAG |
| HK2 | CCGCCGTGGTGGACAAGATA | AGCAGTGATGAGAGCCGCTC |
| mtDNA | CCGCAAGGGAAAGATGAAAGA | TCGTTTGGTTTCGGGGTTTC |
| nuclear DNA | CCCTGTCATGTCCCTTTGTT | GCCACCAGCTCAGTTAAAGG |
| Pgk1 | GCTGTTCTCCTCTTCCTCATC | CCTTTGGTTGTTTGTTATCTGG |
